# Transdermal delivery of vitamin K using dissolving microneedles for the prevention of vitamin K deficiency bleeding

**DOI:** 10.1016/j.ijpharm.2018.02.031

**Published:** 2018-04-25

**Authors:** Aaron R.J. Hutton, Helen L. Quinn, Paul J. McCague, Courtney Jarrahian, Annie Rein-Weston, Patricia S. Coffey, Emily Gerth-Guyette, Darin Zehrung, Eneko Larrañeta, Ryan F. Donnelly

**Affiliations:** a School of Pharmacy, Queen’s University, Belfast, Medical Biology Centre, 97 Lisburn Road, Belfast BT9 7BL, United Kingdom; b PATH, 2201 Westlake Avenue, Suite 200, Seattle, WA 98121, USA

**Keywords:** FTIR, Fourier Transform Infrared Spectroscopy, ICH, International Conference on Harmonisation of Technical Requirements for Pharmaceuticals for Human Use, IM, intramuscular, LEDC, less economically developed country, LoD, limit of detection, LoQ, limit of quantification, MN, microneedle, OCT, optical coherence tomography, PVP, polyvinylpyrrolidone, RH, relative humidity, UK, United Kingdom, UV, ultraviolet, VKDB, vitamin K deficiency bleeding, Dissolving microneedles (MN), Less economically developed countries (LEDCs), Transdermal drug delivery, Vitamin K, Vitamin K deficiency bleeding (VKDB)

## Abstract

Vitamin K deficiency within neonates can result in vitamin K deficiency bleeding. Ensuring that newborns receive vitamin K is particularly critical in places where access to health care and blood products and transfusions is limited. The World Health Organization recommends that newborns receive a 1 mg intramuscular injection of vitamin K at birth. Evidence from multiple surveillance studies shows that the introduction of vitamin K prophylaxis reduces the incidence of vitamin K deficiency bleeding. Despite these recommendations, coverage of vitamin K prophylactic treatment in low-resource settings is limited.

An intramuscular injection is the most common method of vitamin K administration in neonates. In low- and middle-income countries, needle sharing may occur, which may result in the spread of bloodborne diseases. The objective of our study was to investigate the manufacture of microneedles for the delivery of vitamin K. Following microneedle fabrication, we performed insertion studies to assess the microneedle’s mechanical properties. Results indicate that vitamin K in a microneedle array was successfully delivered *in vitro* across neonatal porcine skin with 1.80 ± 0.08 mg delivered over 24 h. Therefore, this initial study shows that microneedles do have the potential to prevent vitamin K deficiency bleeding. Future work will assess delivery of vitamin K in microneedle array *in vivo*.

## Graphical abstract

**Figure:**
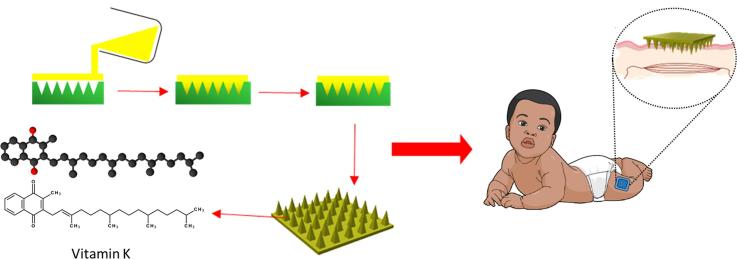

## 1 Introduction

Vitamin K, a fat-soluble vitamin, functions as a cofactor for gamma-carboxylase enzymes within the liver (Ozdemir et al., 2012). This role enables posttranslational conversion of inactive hepatic precursors II, VII, IX, and X into active clotting factors through gamma-carboxylation of glutamic acid residues (Shearer, 1995, Smith et al., 2015). Hepatocyte secretion into the blood signifies the end stage of carboxylation, enabling regulation of the clotting cascade (Smith, et al., 2015).

Due to its fat-soluble properties, vitamin K is largely stored in the liver of adults and children. Only a small amount (approximately 1 µg/kg/day) is required in the diet. Sources of vitamin K include green leafy vegetables, vegetable oils, and cereal grains (Shearer, 1995). However, within neonates, a relatively small amount of vitamin K is found in the liver compared to other fat-soluble vitamins (Kayata et al., 1989). In addition, poor diffusion of vitamin K across the placenta and only very low concentrations of it within breast milk put newborns at risk for vitamin K deficiency bleeding (VKDB), a rare and potentially dangerous bleeding disorder in infants in the first hours to months of life (Anonymous, 1993, Kries et al., 1987, Sutor et al., 1999). As defined by the Paediatric Subcommittee of the International Society on Thrombosis and Haemostasis, VKDB can present itself in three forms: early, classic, and late (Schulte et al., 2014). Early VKDB can present as an intracranial haemorrhage during the first 24 h of birth. This particular form is most common in newborns whose mothers have been prescribed antiepileptics, anticoagulants, or other medicines that interact and prevent the normal functioning of vitamin K (Shearer, 2009). In contrast to early VKDB, the classic form of this condition is largely regarded as idiopathic with the gastrointestinal tract, umbilical region, and skin, which are defined as common bleeding sites. These classic symptoms present themselves between the second and seventh day after birth (Shearer, 2009). Late VKDB has been shown to mainly affect breastfed infants; however, there have been reports of links with hepatobiliary dysfunction alongside other mild abnormalities of the liver (Matsuda et al., 1989, Newman and Shearer, 1998, von Kries et al., 1985). Bleeding due to this form of deficiency is present after the eighth day and up to six months postbirth (Sutor et al., 1999).

Vitamin K prophylaxis can prevent VKDB (Schulte et al., 2014). The World Health Organization recommends that newborns receive a 1 mg intramuscular (IM) injection of vitamin K at birth. Evidence from multiple surveillance studies shows that the introduction of vitamin K prophylaxis reduces the incidence of VKDB (Schulte et al., 2014). Current recommendations support universal prophylaxis due to the lack of predictors for vitamin K deficiency (Shearer, 2009). Despite these recommendations, coverage of vitamin K prophylactic treatment in low-resource settings is limited (Newman and Shearer, 1998).

Oral formulations are available, but the optimal regimen and associated efficacy of oral doses are not well understood. Both oral and IM routes present disadvantages, which may affect their efficacy. Oral vitamin K is administered as three 1 mg doses at intervals of up to 5 weeks (von Kries et al., 1995). Due to the short duration of action of each dose, poor absorption in unknown liver abnormalities, and possible compliance issues, infants may not be protected against late VKDB by oral administration of vitamin K (Sutor et al., 1999). IM administration, on the other hand, involves a single 1 mg dose of vitamin K. This is due to its ability to produce mean plasma levels of vitamin K 20,000 times greater than normal levels, 4 h postinjection (McNinch, 2010, Shearer, 1995). Although effective, a recent survey in a single southeastern state in the United States highlighted the concerns and mindset of expectant parents intending to refuse IM vitamin K prophylaxis (Hamrick et al., 2016). Eighty-three per cent of parents in this study reported awareness of the risks associated with vitamin K refusal; however, only 11% decided to accept IM prophylaxis after discussions with study workers (Hamrick et al., 2016). Reasons for refusal included concerns over injection pain, localised bruising, and toxic ingredients (Hamrick et al., 2016).

A novel delivery mechanism using drug-loaded dissolving microneedles (MNs) has been designed to provide a potential alternative to hypodermic needles (Kaushik et al., 2001). MN arrays consist of micron-scale projections arranged in perpendicular orientation to a baseplate. MNs can encapsulate drug within the baseplate or throughout the entire array (González-Vázquez et al., 2017, McCrudden et al., 2014). Dissolving MN arrays have been extensively used for the delivery of a wide range of compounds, such as small molecules, proteins and nanoparticles (Donnelly et al., 2012, González-Vázquez et al., 2017, Kennedy et al., 2017 Larrañeta et al., 2016a, Larrañeta et al., 2016b, Vora et al., 2017). Upon application to the skin, interstitial fluid causes the MN polymer to dissolve, resulting in the release of the drug. This platform has the ability to create microconduits through the *stratum corneum*, enabling the delivery of drugs into the deeper regions of the skin where they can be absorbed directly into the systemic circulation (Donnelly et al., 2012).

There are significant advantages in facilitating transdermal drug delivery using MNs, one of which is self-application (Bariya et al., 2012, Ripolin et al., 2017). As a result of a micron-scale design, penetration does not reach the dermal layers, meaning application is pain-free (Giudice and Campbell, 2006, Xie et al., 2005). Research has proven that patients show no needle fear, discomfort is reduced, and healing is faster at the injection site (Haq et al., 2009, Kaushik et al., 2001, Xie et al., 2005). Furthermore, as this design of MN is dissolvable, it is limited to one application; thus, it prevents needlestick injury and eliminates sharps disposal requirements.

To date, dissolving MN arrays have been used to deliver hydrophilic drug molecules such as theophylline, caffeine, and lidocaine (Garland et al., 2012). On the other hand, hydrophobic, liquid drugs have yet to be tested within an MN-based delivery platform. The objective of this study was to incorporate vitamin K into a dissolving MN array for neonatal prophylaxis of VKDB. This has the potential benefit of improving acceptance within developed countries and greatly reducing VKDB cases in less economically developed countries (LEDCs).

## 2 Materials and methods

### 2.1 Chemicals

Gantrez® S-97, a copolymer of methyl vinyl ether and maleic acid, was a gift from Ashland, Kidderminster, United Kingdom (UK). Tween® 80 and vitamin K_1_ (MW 450.71) were purchased from Tokyo Chemical Industry UK Ltd., Oxford, UK. Polyvinylpyrrolidone (PVP; MW 40,000 Da) and hyaluronic acid (300 kDa) were purchased from Sigma-Aldrich Company Ltd., Dorset, UK. All other chemicals used were of analytical grade.

### 2.2 Manufacture of dissolving MN arrays

Vitamin K MNs were prepared by adding 2% w/w of vitamin K to 5% w/w Tween® 80, followed by gentle mixing. The appropriate volume of deionised water was then added and mixed to ensure dispersion. A 20% w/w aqueous blend of Gantrez® S-97 was prepared by adding the correct mass to the vitamin K suspension, followed by centrifugation at 2739*g* for 10 min. Approximately 500 mg of formulation was poured into laser-engineered silicone micromolds, composed of 121 (11 × 11) conical holes, 600 µm in depth, with a base width of 300 µm and interspacing of 300 µm. Each array was centrifuged for 15 min at 2739*g*, covered in aluminium foil, and allowed to dry under ambient conditions for 48 h. After 48 h, MN arrays were demoulded and sidewalls removed by a heated scalpel. The mass of each MN array was recorded to determine vitamin K content.

### 2.3 Physical testing of MN arrays

Compression and insertion studies of vitamin K MN arrays were tested using a TA.XT2 Texture Analyser (Stable Micro Systems Ltd., Haslemere, UK). Before compression, needle heights were visualised and measured using a Leica EZ4W stereo microscope. Parafilm M® (Bemis Company Inc., Soignies, Belgium) was made into eight layers to artificially simulate skin (Larrañeta et al., 2014). The MN arrays were then inserted into the polymeric film using the Texture Analyser, at a speed of 1.19 mm/s and force of 32 N, held for 30 s. Previous research works determined that this is the average force applied by human volunteers in MN insertion experiments (Larrañeta et al., 2014, Lutton et al., 2015). Needle height was measured as before to determine compression, expressed as percentage height reduction. To quantify insertion, each layer of Parafilm M® was examined under the microscope and the number of holes counted.

### 2.4 Fourier transform infrared spectroscopy analysis of vitamin K–loaded MN arrays

Four scans (Tween® 80, vitamin K, 20% w/w Gantrez® S-97 with 5% w/w Tween® 80 MN array, and a vitamin K–loaded MN array) were performed using an FTIR (Fourier Transform Infrared Spectroscopy) Accutrac FT/IR-4100 Series (Jasco, Essex, UK) equipped with Diamond MIRacle™ ATR. To obtain each spectrum, an average of 64 scans were recorded in the region of 4000–500 cm^−1^ at a resolution of 4.0 cm^−1^.

### 2.5 Determination of vitamin K recovery from MN arrays

Each vitamin K MN array was placed in 100% ethanol, covered in aluminium foil, incubated at 37 °C, and agitated at 40 rpm. Once fully dissolved, a sample was taken, diluted 100-fold, and assessed using ultraviolet (UV) spectroscopy at wavelength 270 nm. This enabled drug content to be quantified, which was expressed in terms of percentage drug recovery.

### 2.6 Humidity stability testing

The control for this test, relative humidity (RH) 43%, considered to be bench-top humidity, was created using a saturated solution of potassium carbonate (Greenspan, 1977). Comparison was made against RH 86% by creating a saturated solution of potassium chloride (Greenspan, 1977). Three MN arrays were placed into each environment, temperature controlled at 25 °C, and protected from light for 3 days. Physical characterisation and drug recovery were performed at day 0 and day 3.

### 2.7 *In vitro* permeation studies

Vitamin K permeation through dermatomed (350 µm) neonatal porcine skin (previously shown to be predictive of *in vivo* dissolving MN performance^25^) was quantified using Franz diffusion cells. Skin was shaved using a disposable razor and fixed to the Franz cell donor compartment using cyanoacrylate glue. Barrier integrity was confirmed using transepidermal water loss (Vapometer™, Delfin Technologies Ltd, Kuopio, Finland) with the skin/donor compartment sitting atop the filled receiver compartment. The skin/donor compartment set-up was then placed on dental wax covered with aluminium foil to provide support and to confirm MN skin insertion. The MN array was placed in the centre of the skin and gentle pressure applied for 30 s, with a 5.0 g circular stainless steel weight then placed on top. Each receiver compartment was filled with PBS (pH 7.4), thermostatically maintained at 37 ± 1 °C. A metal stir bar was added to provide agitation (600 rpm) before the donor compartment was placed on top and clamped into place. To prevent losses to evaporation, Parafilm M® was placed over each Franz cell before occluding the sampling arm with Blu-Tack™ (Bostik Ltd, Leicester, UK). At each specified time point, 200 µL samples were removed from the receiver compartment using 1 mL syringes attached to 8.0 cm needles and an equal volume of fresh pre-warmed PBS added to replace this. To determine drug content, samples were filtered, diluted appropriately in 100% ethanol and vortexed for 10 s before UV analysis.

### 2.8 Pharmaceutical analysis of vitamin K

Vitamin K was quantified using UV spectroscopy (Epoch Microplate Spectrophotometer with Gen5 software, BioTek, Winooski, VT, USA). Vitamin K has two absorption maxima (248 nm and 270 nm). However, to reduce interference, the longer wavelength was used. Six concentrations were prepared by serial dilution from a 200 µg/mL stock solution, using 100% ethanol as the solvent. Three 200 µL samples of each solution were placed on an acrylic 96 well plate (Corning Incorporated, Kennebunk, USA) prior to UV analysis. Samples containing skin extracts were employed to confirm lack of interference with curve linearity.

Least squares linear regression analysis and correlation analysis provided the coefficient of determination (R^2^), line equation, and residual sum of squares for each calibration plot produced over three consecutive days. Limit of detection (LoD) and limit of quantification (LoQ) were calculated using Eqs. (1), (2), with S notating the slope of the calibration curve and σ the standard deviation of the response.

(1) $$\mathrm{LoD}=\frac{3.3\sigma }{S}$$

(2) $$\mathrm{LoQ}=\frac{10\sigma }{S}$$

Accuracy and relative standard deviation were calculated from both intraday and interday calibration plots to confirm validity. Three vitamin K concentrations (2.5, 5, and 20 µg/mL) within the linear range were chosen for analysis.

### 2.9 Statistical analysis

Data were analysed, where appropriate using the Mann-Whitney *U* test. In each case, *p* < 0.05 was considered to denote significance. Statistical analysis was performed using GraphPad Prism® version 5.0 (GraphPad Software Inc., San Diego, California, USA).

## 3 Results

### 3.1 Manufacture of dissolving vitamin K MN arrays

**Fig. 1:**
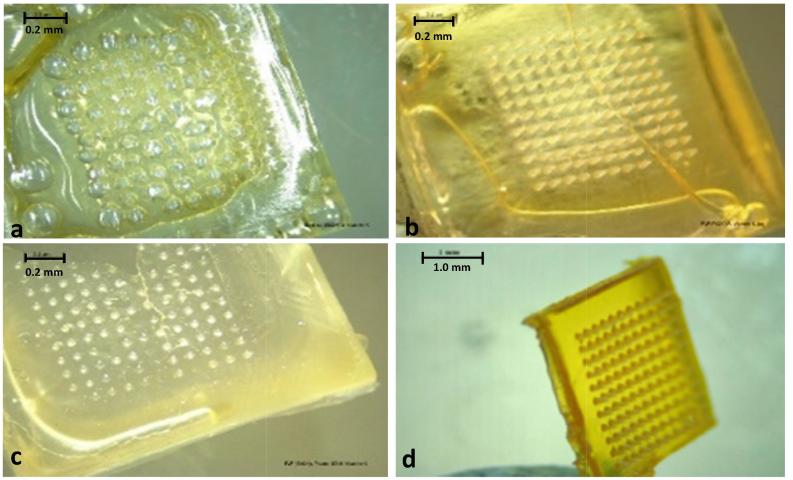
Light microscope images of vitamin K MN formulations. a) MN formulated from 20% w/w Gantrez® S-97 in 100% ethanol produced a large number of bubbles throughout the array. b) MN formulated from 20% w/w PVP in 100% ethanol produced a brittle needle, although all the needles did form. c) MN formulated from 20% w/w PVP in deionised water, containing 5% w/w Tween 80® produced a very brittle needle. Some needles did not form either. d) MN formulated from 20% w/w Gantrez® S-97 and 5% w/w Tween® 80 created a strong array with fully formed needles.

MN arrays were prepared using a variety of polymers and solvents alone or in combination with Tween® 80 (Fig. 1). Hyaluronic acid–based MN formulation (not pictured) produced a very flexible array that was not capable of skin insertion. A dissolving MN formulation based on 20% w/w Gantrez® S-97, 5% w/w Tween® 80, and 2% w/w vitamin K produced fully formed, sharp needles with a strong baseplate. These physical properties enabled further testing on this formulation.

Over the 48-h drying period, a mass loss of 69.6 ± 0.44% was observed because of water evaporation. This increased the vitamin K concentration to 13.2 ± 0.25% w/w. A further 74.3 ± 3.48% mass loss was calculated due to removal of sidewalls. This resulted in an average vitamin K content of 5.14 ± 0.70 mg per array.

### 3.2 Mechanical and humidity stability testing of MN arrays

To investigate the effects of compression, a TA.XT2 Texture Analyser was used. This applied a 32 N uniform force to each MN array for 30 s. This conditions were taken from previous works as they simulate the average force applied by human volunteers when applying MN arrays (Larrañeta et al., 2014, Lutton et al., 2015). Moreover, in a previous work, Larrañeta et al. showed 11 × 11 MN arrays (600 µm needle height) presented the maximum insertion for forces higher than 30 N. Consequently, the selected force of 32 N was the optimum force for the mechanical testing of the array.

**Fig. 2:**
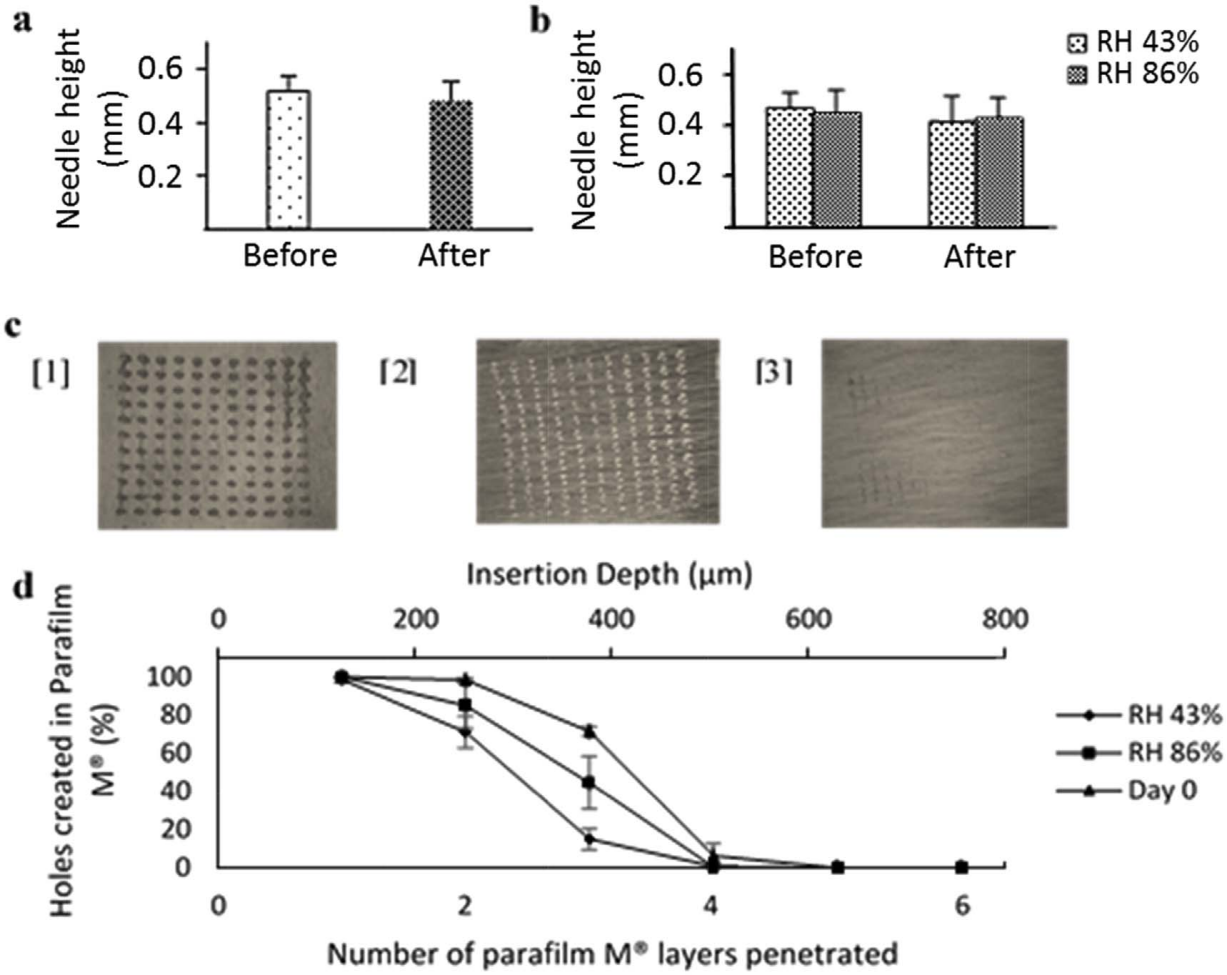
a) Compression analysis of vitamin K MN arrays, formulated from 20% w/w Gantrez® S-97 and 5% w/w Tween® 80, at day 0. Before compression, needle height was 0.517 ± 0.057 mm (means ± SD, *n* = 3). After a 32 N force was applied, needle height was 0.480 ± 0.072 mm. b) Comparison of vitamin K MN arrays subjected to RH 43% or RH 86% for 3 days. At RH 43%, before and after compression, needle height was 0.468 ± 0.065 mm and 0.415 ± 0.103 mm respectively (means ± SD*, n* = 3). RH 86% resulted in needle heights of 0.449 ± 0.093 mm and 0.427 ± 0.086 mm, before and after compression (means ± SD*, n* = 3). c) Images of the first 3 layers of Parafilm M® used to measure insertion of a vitamin K MN array at day 0. Layers 1 and 2 show 100% needle penetration, with 72% needle penetration through the third layer. d) Number of Parafilm M® layers penetrated and percentage holes created in each layer to enable calculation of mean insertion depth for 3 MN conditions.

After compression on day 0, there was no significant difference in needle height following insertion into Parafilm M® (*p* > 0.05). This resulted in a mean height reduction of 7% as shown in Fig. 2a. An estimation of skin depth penetration was assessed using eight layers of Parafilm M®. This insertion study was performed in conjunction with compression analysis. On application of a 32 N force on day 0, more than 20% of the needles penetrated the third layer and less than 20% penetrated the fourth layer as shown in Fig. 2d. As described by Larrañeta et al., penetration is regarded as successful if the number of holes created in each layer is greater than 20% (Larrañeta et al., 2014). Therefore, it can be assumed that the needles lie within the third and fourth layers. Furthermore, as a single Parafilm M® layer is approximately 126 µm thick, the insertion depth of each needle is in the range of 378–504 µm.

Vitamin K MN arrays were subjected to RH 43% or RH 86% for 3 days to test the effect of humidity on MN mechanical properties. Mechanical properties were assessed as detailed previously to allow comparison with MN characteristics on day 0. At RH 43%, there was no significant difference in needle height following compression (*p* > 0.05). This resulted in a needle height reduction of 11% as shown in Fig. 2b. At RH 86%, a height reduction of 5% was determined with no significant difference in needle height following compression (*p* > 0.05). It was noted, however, that MN arrays at RH 43% were brittle in contrast to RH 86%, which produced a softer, more flexible MN array. At RH 43%, insertion studies revealed less than 20% needle penetration into the third layer of Parafilm M®. This resulted in a penetration depth of 252–378 µm. Less than 20% penetration was observed in the fourth layer at RH 86%, showing a penetration depth of 378–504 µm. Although there was reduced needle penetration and height at RH 43% compared to RH 86%, this was not statistically significant (*p* > 0.05).

### 3.3 Pharmaceutical analysis of vitamin K

Quantification of vitamin K was determined using UV spectroscopy. The wavelength used for UV analysis in this study was 270 nm.

Table 1 shows the calibration curve parameters for UV analysis of vitamin K. Linearity of the calibration plot was observed in the range 0.625–20 µg/mL, confirmed by an R^2^ value of 1. The equation of the calibration plot was defined as y = 0.0178x − 0.0001, with LoD and LoQ confirmed as 0.031 µg/mL and 0.093 µg/mL respectively. Interday and intraday variation was expressed in terms of relative standard deviation and accuracy. Three concentrations were chosen to represent high, middle, and low vitamin K concentrations.

**Table 1:** Calibration parameters for quantification of Vitamin K using UV spectroscopy, as represented by coefficient of determination (R^2^), limit of detection and limit of quantification.

Drug	Concentration Range (µg/mL)	R^2^	Equation of Regression Line	LoD (µg/mL)	LoQ (µg/mL)
Vitamin K	0.625–20	1	y = 0.0178x − 0.0001	0.031	0.093

Illustrated in Table 2, interday and intraday accuracy was calculated at 99.2%–99.6% and 99.6%–101.2% respectively, meaning this method is accurate. Precision expresses the closeness of agreement between a series of measurements obtained from multiple sampling of the same sample under the same conditions. Relative standard deviation for both intraday and interday analysis was found to be low, proving that this method is precise and reproducible.

**Table 2:** Determination of accuracy and precision for the quantification of Vitamin K using UV spectroscopy in 100% ethanol.

### 3.4 FTIR analysis of vitamin K–loaded MN arrays

**Fig. 3:**
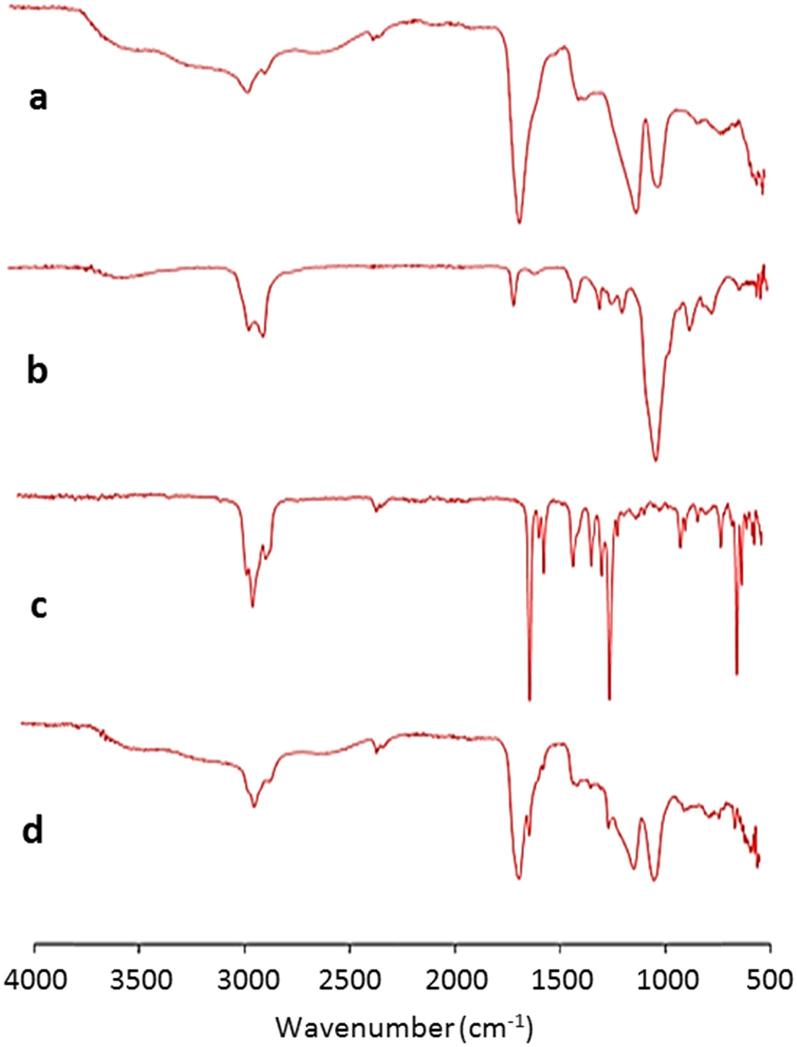
FTIR analysis showing a) Dissolving MN array containing 20% w/w Gantrez® S-97 and 5% w/w Tween® 80 formulated in deionised water. b) Tween® 80. c) Vitamin K. d) Dissolving vitamin K MN array containing 20% w/w Gantrez® S-97, 5% w/w Tween® 80 and 5.14 ± 0.7 mg vitamin K.

**Fig. 4:**
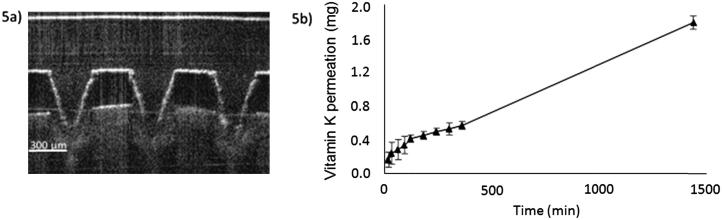
a) Optical coherence tomography image of a dissolving vitamin K MN array following manual insertion into neonatal porcine skin. b) *In vitro* cumulative permeation profile of vitamin K across dermatomed 350 µm neonatal porcine skin using a dissolving MN prepared from aqueous blends containing 20% w/w Gantrez® S-97 and 5% w/w Tween® 80 over 24 h (means ± SD, *n* = 3).

FTIR analysis was performed to assess interactions between vitamin K MN components (Fig. 3). As exploited in swellable MN arrays, there is potential esterification between Gantrez® S-97 acid groups and the hydroxyl groups of Tween® 80 (Donnelly et al., 2014b). However, in this case, no crosslinking occurred as MN fabrication was performed at room temperature. This can be seen in the FTIR spectra as the Gantrez carbonyl peak (1600–1700 cm^−1^) did not show any change (Fig. 4c & d) (Larrañeta et al., 2018, Larrañeta et al., 2015, Larraneta et al., 2018). Visual analysis of each spectrum showed no shift in peaks, proving there are no interactions between MN components.

### 3.5 Determination of vitamin K recovery from MN arrays

Day 0, RH 43% and RH 86% vitamin K MN arrays were recovered to levels above 90% after 24 h in 100% ethanol. The average amount of vitamin K recovered at day 0 was 90.3 ± 4.4%. After 3 days, a higher percentage recovery was determined, with RH 43% and RH 86% producing 96.3 ± 3.2% and 93.5 ± 4.6% respectively. Comparing these results, there is no statistical difference between each condition in relation to drug recovery (*p* > 0.05).

### 3.6 *In vitro* permeation studies

MN insertion into neonatal porcine skin was confirmed before proceeding to an *in vitro* delivery study. MN arrays were inserted into full-thickness (1 mm) porcine skin by applying constant finger pressure for 30 s. Optical coherence tomography (OCT) using an EX1301 OCT microscope (Michelson Diagnostics, Kent, UK) enabled visual assessment of needle insertion. As shown in Fig. 4a, MN arrays had the ability to bypass the *stratum corneum* and reach the dermal layers.

Vitamin K was detected in the Franz receiver compartment by UV analysis after 15 min. As illustrated in Fig. 4b, permeation of vitamin K through neonatal porcine skin occurred throughout the 24-h experiment, with MN arrays delivering 1.80 ± 0.08 mg of vitamin K during this period. As each MN array contains 5.14 ± 0.70 mg of vitamin K, approximately 35% of the contained drug was delivered across the porcine skin in 24 h.

## 4 Discussion

Research in the field of transdermal drug delivery has shown that hydrophobic drugs do not possess the ideal characteristics for skin permeation. A previous study by Lopes et al. highlighted this principle by investigating the delivery of vitamin K using a monoolein-based nanodispersion (Lopes et al., 2007). The results proved that topical administration does not deliver the amount of vitamin K required to alter systemic levels and affect blood coagulation (Lopes et al., 2007). This was due to its low penetration through the *stratum corneum* and the viable epidermis (Lopes et al., 2007).

The data presented here represent the first time vitamin K has been delivered *in vitro* to a sufficient level using a dissolving MN array. The delivery system presented in this study is designed to breach the *stratum corneum* in a pain-free manner, such that the hydrophilic properties required for passive diffusion through the skin are negated.

The ability to achieve a high concentration of vitamin K in the plasma over a short time frame is exploited in IM delivery. Although an injection using this route is reliable and effective, the necessity for a needle poses potentially serious health risks (Haq et al., 2009). In the UK, there are reports of more than 100,000 needlestick injuries in hospitals each year (Anonymous). Injuries of this nature are effectively undocumented in LEDCs; however, a lack of funding and health education in LEDCs is widely believed to result in a greater annual number of injury and needle reuse cases than in developed countries (Anonymous). An assessment by the World Health Organization states that unsafe practice and needle reuse have contributed to 37.6% of hepatitis B, 39% of hepatitis C, and 4.4% of HIV cases worldwide (Prüss-Üstün et al., 2003). Furthermore, in LEDCs, poor disposal systems result in needles residing in open dumps and vacant land, presenting significant danger to “rag scavengers” (Medina, 2010). It is estimated that, in LEDCs, up to 2% of the population survive by scavenging (Medina, 2010). Consequently, unsafe disposal can result in needlestick injury to these vulnerable members of society, hereby increasing the inherent risk of cross-infection of bloodborne pathogens. In contrast, a dissolving vitamin K MN only permits a once-off application, preventing reinsertion into a different individual and eliminating sharps disposal requirements and the risk of cross-contamination.

Dissolving MN arrays are composed of approximately 5%–10% w/w water in the dried state. This presents issues with the incorporation of vitamin K, a water-insoluble vitamin. Vitamin K is soluble in ethanol, so initially this was the solvent used during MN manufacture. This resulted in a large number of bubbles throughout the MN array due to rapid solvent evaporation. A polysorbate-based formulation using Tween® 80 was subsequently devised to aid the solubilisation of vitamin K in water. As a surfactant, this produced a spherical micelle with a hydrophilic surface and hydrophobic core. Due to its hydrophobicity, vitamin K concentrated within the core of Tween® 80. Several polymers were considered. However, PVP-based MN arrays were too brittle and hyaluronic acid MN arrays were too flexible; neither were capable of penetrating the *stratum corneum*. A formulation based on Gantrez® S-97 created a MN array with sufficient strength for insertion. IR analysis was used to display no-component interactions within this formulation. Compression analysis showed that needle height was only reduced by 11%, with all needles remaining intact on the baseplate. Insertion studies using Parafilm M® as an artificial skin model indicated that these MN arrays can penetrate to a depth between 378 and 504 µm. Following this, MN insertion into full-thickness porcine skin was confirmed using OCT imaging. *In vitro* skin permeation of vitamin K increased over the course of the experiment with 1.80 ± 0.08 mg of drug released after 24 h. As mentioned previously, an IM injection delivers 1 mg of vitamin K into each patient. Therefore, this study demonstrates the potential of a 0.49 cm^2^ MN patch delivering a therapeutic dose of vitamin K as prophylaxis for VKDB in newborn babies. The delivered dose is equivalent to approximately 35% of drug permeating through the skin. Although there was visible drug residue on the upper surface of the neonatal porcine skin after 24 h, this could safely be removed and disposed appropriately. Taking into account the hydrophobic nature of vitamin K it is likely that a significant amount of the drug was retained within the SC. However, we believe that the drug wastage is not significant in this case considering all the advantages of the MN system over conventional IM injections.

The dissolving nature of this MN array formulation will result in the deposition of Gantrez® S-97 within the skin. Gantrez® S-97 is a nondegradable polymer approved by the US Food and Drug Administration with widespread use within the cosmetic and pharmaceutical industry; however, its large molecular weight (1500 kDa) is above the threshold for glomerular filtration, which could result in polymer accumulation in the tissue upon repeat administration (Donnelly et al., 2014a, Jones et al., 2016, Quinn et al., 2016). Although Gantrez® S-97 polymer has low toxicity, accumulation could result in erythema or granuloma formation (Quinn et al., 2016). As mentioned previously, this MN array is designed to administer a once-off dose of vitamin K. Therefore, it is predicted that accumulation is unlikely to have any unfavourable effects on the health of neonates.

The effects of humidity on vitamin K MN arrays were assessed by exposing the MN arrays to RH according to ICH guidelines on stability testing of new drug substances and products prior to compression and insertion studies. These guidelines recommend stability testing protocols for climatic Zone I, II, III, and IV to minimize different storage conditions of submission of a global dossier. An equal number of MN arrays were subjected to RH 43% and RH 86% for 3 days. Mechanical testing revealed that RH 43% produced a brittle array in contrast to a more flexible array produced at RH 86%. The brittle nature at RH 43% could be a result of further water evaporation, in contrast to RH 86%, in which water could be absorbed into the MN array, hereby increasing the plasticising effect. The data presented show that transport and storage environments must be highly controlled to maintain the stability of this formulation. Vitamin K is also degraded over time by UV light; this must be considered throughout the development process. With this in mind, future work will involve further stability studies based on temperature and light intensity in addition to *in vivo* drug release testing.

## 5 Conclusion

This study has demonstrated that it is possible to incorporate vitamin K into a dissolving MN array. The formulation chosen enabled successful drug delivery *in vitro* over 24 h. *In vivo* testing to assess safety and pharmacokinetics in a rat animal model will now be performed alongside further investigation into the stability and storage requirements of the formulation.
